# Assessing differences in surgical outcomes following emergency abdominal exploration for complications of elective surgery and high-risk primary emergencies

**DOI:** 10.1038/s41598-022-05326-4

**Published:** 2022-01-25

**Authors:** Woubet Tefera Kassahun, Jonas Babel, Matthias Mehdorn

**Affiliations:** Faculty of Medicine, Clinic for Visceral, Transplantation, Thoracic and Vascular Surgery, University Hospital of Leipzig, Liebig Strasse 20, 04103 Leipzig, Germany

**Keywords:** Outcomes research, Medical research, Risk factors

## Abstract

Irrespective of its etiology, emergency surgical abdominal exploration (EAE) is considered a high-risk procedure with mortality rates exceeding 20%. The aim of this study was to evaluate differences in outcomes in patients who required EAE due to complications of complex elective abdominal procedures and those who required EAE due to high-risk primary abdominal emergencies. Patients undergoing EAE for acute surgical complications of complex abdominal elective surgical procedures (N = 293; Elective group) and patients undergoing EAE for high-risk primary abdominal emergencies (N = 776; Emergency group) from 2012 to 2019 at our institution were retrospectively assessed for morbidity and mortality. Postoperative complications occurred in 196 patients (66.94%) in the elective group and 585 patients (75.4%) in the emergency group. The relatively low complication burden in the elective group was also evidenced by a significantly lower comprehensive complication index score (54.00 ± 37.36 vs. 59.25 ± 37.08, *p* = 0.040). The in-hospital mortality rates were 31% (91 of 293) and 38% (295 of 776) in the elective and emergency groups, respectively. This difference between the two groups was statistically significant (*p* = 0.035). In multivariate analysis, age, peripheral artery disease, pneumonia, thromboembolic events, ICU stay, ventilator dependence, acute kidney failure and liver failure were independent predictors of mortality. Our data show that patients undergoing EAE due to acute complications of major elective surgery tolerate the procedure relatively well compared with patients undergoing EAE due to primary high-risk abdominal emergencies.

## Introduction

Despite continued efforts to improve preoperative patient management and operative techniques, complex elective abdominal surgical procedures still place patients at risk of postoperative complications that sometimes require emergency surgical intervention. These patients are at increased risk of mortality and prolonged hospital stay^1^.

Similarly, irrespective of its etiology, emergency surgical abdominal exploration (EAE) for primary major abdominal emergencies is considered a high-risk procedure with mortality rates exceeding 20%^2–4^. In addition, these patients experience a complication rate nearly triple the rate in elective surgery^5^.

Previous studies have helped identify primary emergency surgery patients as a unique subset of patients requiring extensive surgical care^5–7^. Similarly, there have been several studies reporting procedure-specific surgical complications of complex elective surgical procedures and their outcomes^8–11^. However, little is known regarding differences in outcomes between those patients who required surgery due to acute surgical complications of complex elective abdominal procedures and those who required surgery due to primary high-risk abdominal emergencies.

Therefore, the aim of this study was to evaluate the differences in outcomes between these two groups and provide insight into the risk factors contributing to those differences. We hypothesized that given the standard preoperative care provided by a multidisciplinary team in preparation for elective surgery, patients undergoing EAE for complications of complex elective surgical procedures would have favorable outcomes compared to those undergoing EAE for primary high-risk emergencies.

## Methods

**Figure 1:**
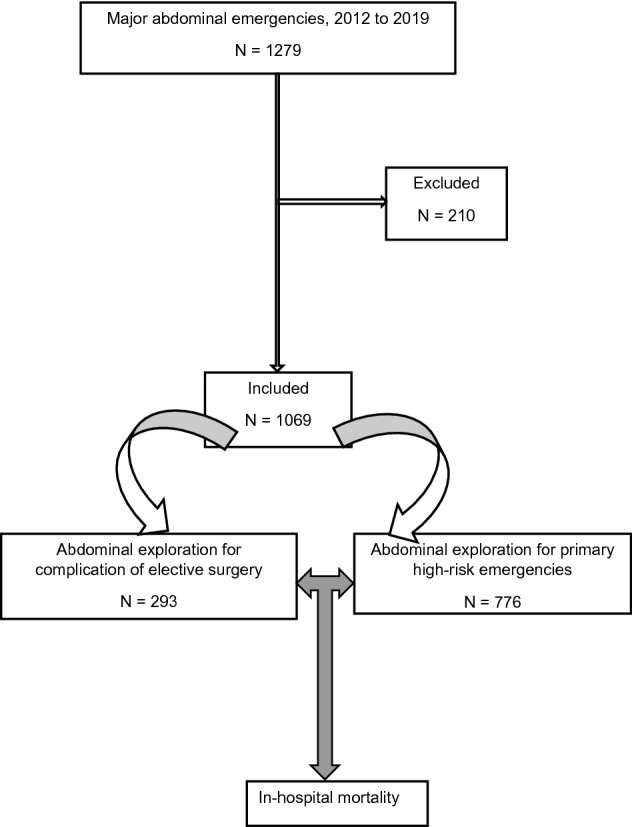
Flow chart of studied patients.

From January 2012 to July 2019, 1279 consecutive patients underwent EAE due to major abdominal emergencies at the Division of Visceral, Transplantation, Thoracic and Vascular Surgery, Department of Surgery, Leipzig University Hospital. All patients who underwent operations due to complications of complex abdominal elective surgical procedures (N = 293; Elective group) and those who underwent operations due to high-risk primary abdominal emergencies (N = 776; Emergency group) during the 7-year period were identified from our database (Fig. 1).

EAE was defined as surgical abdominal exploration that had to be performed as soon as possible after admission or after the onset of related clinical symptoms in a patient aged 18 years and older due to an unscheduled abdominal emergency in an effort to establish a surgically amenable focus. The decision to operate on was made on the basis of clinical and radiologic findings. As the definitive surgical diagnosis and extent of surgery could not be ascertained preoperatively in all instances, the definitive diagnosis and the extent of the surgical procedure were established during abdominal exploration. Because those patients with complications of elective surgery had already undergone laparotomy for the elective diagnosis and were brought back to the operating room for confirmed or suspected complications after the elective procedure, we felt that using the widely accepted term emergency laparotomy (EL) may be inaccurate. Therefore, for the purpose of this study, we use the direct and simple term EAE, which is common for both groups throughout. This avoids confusion about the correct term and allows us to discuss the same procedure with confidence. Based on this definition, we classified patients into two groups: EAE for complications of elective surgery (elective group) and EAE for primary abdominal emergencies (emergency group).

High-risk abdominal emergencies were defined as those that had an anticipated high risk of in-hospital death due to septic and/or hemorrhagic complications and required emergency surgery^6,7,12^. Given our primary focus on evaluating surgical mortality, we limited the study population to patients who underwent emergency procedures that had rates of death of more than or equal to 10%. Almost all patients in the present study were included in this category (Table S2, supplemental).

Patients who underwent EAE for minor emergencies such as cholecystectomies and appendectomies were excluded from this analysis, as were those who underwent laparotomies for complications related to organ transplantation and blunt and penetrating multiple trauma. Patients with minor emergencies were excluded from our study because the low rates of morbidity and mortality, combined with the large number of procedures, could have resulted in misleadingly low overall morbidity and mortality rates. The risk of complications requiring reoperation and death associated with these procedures is extremely low and has been addressed previously in other studies^13,14^. We excluded patients with organ transplantation and multiple trauma from this study because these patients make up a patient population with a different risk profile for complications and mortality. For example, organ transplant patients have an additional risk of complications related to inevitable immunosuppressive medication to protect the transplanted organ from rejection. Similarly, patients with multiple trauma often have other injuries in addition to abdominal trauma, such as cardiopulmonary, vascular or musculoskeletal injuries, that may require surgery simultaneously, increasing the risk of complications and mortality.

With these exclusions, the present study was based on 1069 patients who had EAE and underwent 1 of 14 abdominal procedures depicted in a supplemental table (S2).

Patients who underwent EAE for complications of elective surgery were considered as having had a postoperative complication if that complication was not the acute abdominal event causally related to the index emergency procedure and occurred during and/or after the index procedure.

Data about patient clinical history, demographics, preoperative evaluations, hospitalization, and in-hospital outcomes were obtained from medical records.

In preparation for elective surgery, all patients were evaluated by a multidisciplinary team. Surgical procedures were performed using standard techniques after abdominal exploration was undertaken to identify and confirm the intraoperative diagnosis. Standard postoperative care was applied in all cases.

For the purposes of this study, primary elective major surgical procedures were categorized as follows: (1) hepatobiliary and pancreatic ([HBP] n = 124), (2) upper gastrointestinal ([UGI] n = 50), (3) lower gastrointestinal ([LGI] n = 81), and (4) miscellaneous (n = 38). We included patients after complex high-risk procedures that required substantial technical skill and longer operative times.

The American Society of Anesthesiologists (ASA) class^15^ was used to reflect comorbid conditions, such as cardiopulmonary and renal disease, in all patients. Based on the Clavien-Dindo classification of complications^16^, the comprehensive complication index (CCI) was used to reflect the overall burden of postoperative complications in all patients with one or more postoperative complications.

The major outcome measure studied was the in-hospital mortality rate after EAE. To support future risk assessment, we identified the predictors of mortality.

All deaths after surgery within the first hospital stay, without the patient being discharged from the hospital, were considered to represent in-hospital mortality.

### Statistical analysis

The results of patients who underwent EAE for complications of complex elective procedures were compared with the results of patients who underwent EAE for high-risk major abdominal emergencies. The chi-square test or Student *t* test (2-tailed) was used for univariate comparisons, as appropriate. Multiple logistic regression analysis was used to incorporate all the explanatory variables in the same model, as described in detail in a previous publication^4^. All data are presented as the percentage of patients, means ± standard deviations (SDs), or medians with ranges (Rs). Relative risks (RRs) or odds ratios (ORs) and their 95% confidence intervals (CIs) are presented for each estimate. Differences were considered significant at *p* ≤ 0.05. All analyses were performed using SPSS software, version 26 (IBM Corporation, USA).

The study was approved by the institutional review board of the medical faculty of the University of Leipzig. As this was a retrospective study, the need for informed consent was waived by the ethical committee of the medical faculty of the University of Leipzig. We confirm that all methods are carried out in accordance with the relevant guidelines and regulations of the ethics committee.

## Results

### Baseline patient characteristics and coexisting conditions.

Overall, more male patients (651, 61%) than female patients underwent EAE, and the distribution of sex among patients in the elective group compared with those in the emergency group was significantly different.

The age distribution, distribution of ASA classes, BMI values and the overall prevalence of coexisting conditions did not differ between the two groups. Table 1

**Table 1:** Baseline patient characteristics.

Variable	Elective group	Emergency group	*p* value
	N = 293	N = 776	
Male-sex	209 (71.3)	442 (57.0)	< 0.0001
Age, years, mean ± SD	63.31 ± 13.49	65.29 ± 16.83	0.071
BMI, mean ± SD	27.04 ± 7.70	26.36 ± 6.52	0.152
Comorbid conditions (COCs)	230 (78.5)	593 (76.4)	0.471
COCs per patient, mean ± SD	4.88 ± 2.39	5.32 ± 3.02	0.029
ASA ≥ 3	230 (79.6)	592 (79.6)	1
**Major COCs**			
Hypertension	205 (70.0)	503 (64.8)	0.113
Cardiac arrhythmias	46 (15.7)	228 (29.4)	< 0.0001
Coronary artery disease	47 (16.0)	133 (17.1)	0.669
Chronic heart failure	35 (11.9)	148 (19.1)	0.006
Peripheral arterial disease	39 (13.3)	199 (25.6)	< 0.0001
Chronic kidney disease	41 (14.0)	135 (17.4)	0.562
Diabetes mellitus	73 (24.9)	185 (23.8)	0.714
COPD	40 (13.7)	116 (14.9)	0.592
**Primary elective procedures**			
Hepatobiliary and Pancreatic	124 (42.3)	–	–
Upper GI	50 (17.1)	–	–
Lower GI	81 (27.6)	–	–
Miscellaneous	38 (13)	–	–
OT, minutes, mean ± SD	301 ± 152.41	–	–
TIE, days, mean ± SD	8.03 ± 8.31	–	–

Numbers in bracket indicate values presented in n (%) by group unless noted otherwise.

*BMI* body mass index, *SD* standard deviation, *COPD* chronic obstructive pulmonary disease, *ASA* the American society of anesthesiologists physical status classification, *COCs* comorbid conditions, *OT* operative time for the elective procedure, *TIE* time interval from the elective procedure to the first emergency laparotomy.

Patients who underwent EAE for high-risk abdominal emergencies had more coexisting conditions per patient.

In both groups, the most common coexisting condition was hypertension, followed by cardiac disorders and diabetes mellitus. Atrial fibrillation, chronic heart failure and peripheral artery disease (PAD) were less prevalent in the elective group than in the emergency group (*p* < 0.05).

### Primary indications for surgery and index procedures.

The proportions of patients with a diagnosis of perforation, mesenteric ischemia or bowel obstruction were greater among the primary emergency group than among the elective group. Table S1 (supplemental).

However, abdominal sepsis, defined as the presence of an abscess, anastomotic leak, fistula, ischemia, perforation, or toxic colitis, was the most common emergency diagnosis among patients in both groups (61% of the elective group patients and 60% of the emergency group patients) (Table 2).

**Table 2:** Primary indications for emergency surgery and surgical procedures by group.

	Elective group	Emergency group
	N = 293	N = 776
**Surgical emergency**		
Abdominal sepsis	178 (60.8)	466 (60.0)
Bowel obstruction	17 (5.8)	208 (26.8)
Hemorrhage	44 (15.0)	58 (7.5)
Burst abdomen	43 (14.7)	–
Miscellaneous	11 (3.8)	44 (5.7)
**Index procedure**		
Small bowel resection	18 (6.1)	109 (14.1)
Colon resection	36 (12.3)	203 (26.14)
Closure of viscus organ	49 (16.7)	128 (16.5)
Lysis of adhesions	99 (33.8)	99 (12.8)
Control of hemorrhage	34 (11.6)	48 (6.2)
Simultaneous MPs	32 (10.9)	120 (15.4)
Miscellaneous procedures	25 (8.5)	69 (8.9)

Numbers in bracket indicate values presented in n (%).

*MPs* multiple procedures.

Of those who underwent an operation due to complications of elective surgery, 124 patients had primary procedure codes for major HBP procedures, 50 patients had primary procedure codes for upper-GI procedures, 81 patients had primary procedure codes for lower-GI procedures, and 38 patients had procedure codes for miscellaneous procedures. The average time from the elective procedure until the first EAE was 8 days.

With few exceptions, the distribution of index procedures at the time of the emergency differed significantly between the two groups. Bowel resection was relatively less common in the elective group (18.4%). This finding was 2 times (40.2%) more common in the emergency group. However, extensive adhesiolysis with lavage was performed more frequently in the elective group (34%) than in the emergency group (12.8%).

### Incidence of postoperative complications and in-hospital mortality

Postoperative complications (after the index emergency procedure) occurred in 196 patients (66.9%) in the elective group and 585 patients (75.4%) in the emergency group. The low overall complication burden in the elective group was also evidenced by a significantly lower CCI score (54.00 ± 37.36 vs. 59.25 ± 37.08, *p* = 0.04). Common complications were intraabdominal hemorrhage, including intraabdominal hemorrhage, anastomotic or bile leakage, and liver failure in the elective and emergency groups, respectively. Cardiovascular, pulmonary and renal complications were also common, including thromboembolism, pneumonia and acute renal failure requiring dialysis. Surgical site infections were the most common complications, occurring in 47.4% of the patients in the elective group and 37.9% of the patients in the emergency group (Table 3).

**Table 3:** Outcomes.

Variable	Elective group	Emergency group	*p* value
	N = 293	N = 776	
Complications overall	196 (66.9)	585 (75.4)	0.005
Multiple complications	142 (48.6)	495 (63.8)	< 0.0001
Complications PP, mean ± SD	3.92 ± 3.30	5.41 ± 3.53	0.022
CCI, mean ± SD	54.00 ± 37.36	59.25 ± 37.08	0.040
**Surgical complications**	153 (52.2)	417 (53.7)	0.657
Hemorrhage	31 (10.6)	209 (26.9)	< 0.0001
Surgical site infection	139 (47.4)	294 (37.9)	< 0.005
Anastomotic leak	48 (16.4)	119 (15.3)	< 0.674
**Medical complications**	164 (56.0)	428 (55.2)	0.810
Pneumonia	66 (22.5)	236 (30.4)	0.011
TEEs	29 (9.9)	149 (19.3)	< 0.0001
Liver failure	50 (17.1)	190 (24.5)	0.010
Acute renal failure	64 (21.8)	281 (36.4)	< 0.0001
Respiratory compromise	108 (36.9)	354 (45.6)	0.010
Reoperation	117 (40.1)	309 (40.2)	0.961
Reoperation PP, mean ± SD	2.32 ± 1.75	2.0 ± 1.50	0.048
ICU	130 (44.4)	620 (79.9)	< 0.0001
ICU-LOS, days, mean ± SD	19.91 ± 23.86	12.61 ± 17.85	< 0.0001
ICU-LOS, days, median (IR)	12 (1–174)	6 (1–135)	
MV	172 (58.7)	461 (59.4)	0.834
DMV, hours, mean ± SD	215.85 ± 295.63	202.56 ± 313.76	0.688
DMV, hours, median (R)	72.5 (1–3747)	64 (1–1865)	
In-hospital mortality	91 (31.1)	295 (38.0)	0.035
TSD, days, mean ± SD	24.26 ± 31.17	14.44 ± 23.42	0.004
TSD, days, median (R)	11.0 (1–92)	4 (1–173)	
LOS, days, mean ± SD	40.00 ± 29.91	22.13 ± 23.50	< 0.0001
LOS, days, median (R)	34 (3–200)	14 (1–200)	

*PP* per patient; surgical site infection is defined as being contained within the skin or subcutaneous tissue (superficial), or involving the muscle and /or fascia (deep); acute renal failure was considered if it required dialysis, *CCI* the comprehensive complication index, *TEEs* thromboembolic events, *ICU* intensive care unit requirement, *MV* mechanical ventilation defined as ventilation at any time during hospitalization and applies for all patients who required ventilation beyond the operation room, *DMV* duration of mechanical ventilation, *TSD* time interval from the index emergency procedure to death, *LOS* length of hospital stay defined as the time from the date of the initial admission to the date of discharge, transfer to external services, or death, which ever came first.

The proportion of reoperated patients was similar in both groups. One hundred seventeen patients (40.1%) in the elective group and 309 patients (40.2%) in the emergency group underwent reoperations for re-examination or further complications. However, there were more reoperations per patient in the elective group than in the emergency group (2.32 ± 1.75 vs. 2.0 ± 1.50, *p* = 0.048).

The median length of hospital stay (LOS) and the median interval from the index emergency procedure to death were significantly longer for those undergoing EAE due to complications of elective procedures than for those undergoing EAE due to primary major abdominal emergencies (34 vs. 14 days and 11 vs. 4 days, respectively, *p* < 0.05).

**Figure 2:**
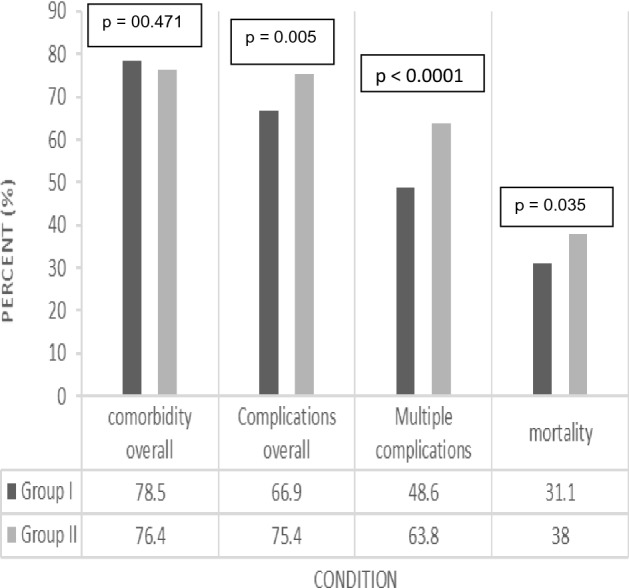
Coexisting conditions and outcomes by group. Group I = Elective group. Group II = Emergency group.

The in-hospital mortality rates were 31.1% (91 of 293) and 38% (295 of 776) in the elective and emergency groups, respectively. This difference between the two groups was statistically significant (*p* = 0.035) (Fig. 2).

In over 80% of the patients who died in both groups, postoperative sepsis with multisystem organ failure was the cause of death.

### Predictors of mortality

To determine which factors were important for predicting in-hospital mortality in patients who underwent surgery due to complications of elective surgery or primary emergencies, a multivariate analysis was performed. Our models were based on backward stepwise logistic regression. Our final model included 20 variables as potential predictors of mortality (Table 4).

**Table 4:** Relative risk (RR) for mortality for all patients (univariate analysis).

Variable	RR (95% CI)	*p* value
Age ≥ 70 years	2.73 (2.24–3.16)	< 0.0001
ASA ≥ 3	6.09 (3.71–9.98)	< 0.0001
Hypertension	1.40 (1.16–1.69)	< 0.0001
Heart failure	1.37 (1.14–1.64)	0.001
Coronary artery disease	1.37 (1.15–1.65)	0.001
Atrial fibrillation	1.71 (1.46–1.99)	< 0.0001
PAD	2.06 (1.77–2.38)	< 0.0001
COPD	1.32 (1.09–1.60)	0.008
CKD	1.52 (1.28–1.81)	< 0.001
OAC	1.44 (1.23–1.68)	< 0.0001
ICU	26.37 (8.56–81.21)	< 0.0001
Hemorrhage	2.49 (2.16–2.88)	< 0.0001
BPT	2.84 (2.44–3.30)	< 0.0001
Anastomotic leakage	1.71 (1.45–2.01)	< 0.0001
Pneumonia	2.70 (2.16–3.14)	< 0.0001
Thromboembolic events	2.50 (2.18–2.87)	< 0.0001
Acute renal failure	7.74 (6.07–9.86)	< 0.0001
Liver failure	5.53 (4.74–6.45)	< 0.0001
VD	12.61 (8.17–19.44)	< 0.0001
Reoperation	2.16 (1.83–2.55)	< 0.0001
Group emergency	1.09 (1.01–1.17)	0.035

*RR* relative risk, *CI* confidence interval, *VD* ventilator dependence (ventilation longer than 24 h at any time during hospitalization), *PAD* peripheral arterial disease, *COPD* chronic obstructive pulmonary disease, *OAC* oral anticoagulation, *BPT* blood product transfusion, *CKD* chronic kidney disease, *ICU* intensive care unit stay.

Factors that were statistically significant predictors of mortality in all patients undergoing EAE were age (OR, 2.30; *p* = 0.001), PAD (OR, 2.42; *p* = 0.001), ICU stay (OR, 5.38; *p* = 0.024), pneumonia (OR, 2.11; *p* = 0.002), thromboembolic events (OR, 2.33; *p* = 0.002), acute kidney failure (OR, 7.06; *p* < 0.0001), liver failure (OR 18.80; *p* < 0.0001), and ventilator dependence (OR, 6.06; *p* < 0.0001) (Table 5).

**Table 5:** Multivariate analysis: predictors of in-hospital mortality after adjusting for risk factors

Risk factor	OR (95% CI)	*p* value
Age	2.30 (1.38–3.81)	0.001
PAD	2.42 (1.41–4.16)	0.001
ICU	5.38 (1.25–23.2)	0.024
Pneumonia	2.11 (1.30–3.38)	0.002
TEEs	2.33 (1.35–4.01)	0.002
Liver failure	18.80 (9.26–38.00)	< 0.0001
Acute renal failure	7.06 (4.26–11.70)	< 0.0001
VD	6.06 (3.03–12.12)	< 0.0001

Adjusted for age, ASA, hypertension, atrial fibrillation, CAD, PAD, COPD, CKD, OAC, ICU, chronic heart failure, bleeding events, BPT, anastomotic leaks, pneumonia, thromboembolic events, liver failure, acute renal failure, URL, VD, and Group emergency. Each of the 21 risk factors were associated with significant risk for in-hospital mortality in univariate analysis.

## Discussion

The outcomes after EAE are poor, with disproportionately high mortality rates ranging from 15 to 44%^3,6,17,18^. Approximately one out of five EAE procedures are performed as a result of postoperative complications^6^.

The purpose of the present study was to analyze the differences in outcomes between patients who underwent EAE due to complications of elective surgery and those who underwent EAE due to primary high-risk abdominal emergencies.

To our knowledge, this is the first study to report the differences in outcomes after EAE in these two patient populations on a large scale.

Patients who underwent surgery due to emergency complications of elective abdominal surgical procedures had lower morbidity and all-cause in-hospital mortality rates but a longer LOS than patients who underwent surgery due to primary major abdominal emergencies. The most important predictors of mortality were (1) age, (2) peripheral artery disease, (3) pneumonia, (4) thromboembolic events, (5) acute renal failure, (6) liver failure, (7) ICU stay, and (8) ventilator dependence.

Poor outcomes are associated with complications after high-risk surgery, and among patients who die, roughly two-thirds have more than one complication^1,19,20^. In agreement with this, in our study, more patients in the primary emergency group had multiple complications than in the elective group, and almost all patients who died after surgery in the high-risk primary emergency group had multiple complications. Thus, the development of multiple complications may have incrementally affected patients in the high-risk emergency group more than patients in the elective group regarding in-hospital mortality. Acute kidney failure and postoperative liver dysfunction portended a particularly poor outcome in our composite cohort that can be mainly explained by multiorgan failure. Sepsis with multiple organ failure was the primary cause of death in both groups, accounting for 82.4% (75 of 91 deaths) of deaths in the elective group and 83.7% (247 of 295 deaths) of deaths in the emergency group. This provides evidence that medical complications have a far stronger association with adverse outcomes and is in agreement with the results of other studies that attributed high mortality to sepsis-related postoperative medical complications^5,21,22^.

Mortality was markedly high in both groups, which is a reflection of high-risk abdominal events. In particular, cases of intestinal ischemia, viscous organ perforation, hemorrhage and anastomotic leakage are considered high-risk conditions^6,23^.

Patients who undergo elective major abdominal surgery are appropriately assessed to identify modifiable risk factors, such as cardiovascular, pulmonary, and renal factors, that can be addressed before surgery. In the primary emergency setting, the opportunity to do so is limited. It is likely that risk-reduction strategies appropriate for a primary emergency procedure population will differ from those applicable to the population undergoing elective procedures. Consequently, the standard preoperative care provided by a multidisciplinary team in preparation for the elective surgical procedure may have contributed to the relatively favorable in-hospital mortality rate in the elective group when compared with the emergency group. Indirect support for this positive impact of preoperative management on the outcome after surgery for complications is provided by the finding that both groups had comparable age distributions, BMI values, ASA classes, and overall prevalence of coexisting conditions.

A number of mechanisms have been proposed to explain outcome differences after surgery. One of the potential factors at the patient level is age^5^. Our study found that advanced age is an independent risk factor for mortality across the entire emergency population.

The majority of patients aged 70 years or older (105 of 115 in the elective group vs. 301 of 360 in the emergency group) had 1 or more complications following surgery, and the surgery-related risk of mortality was also strikingly high (39.1% vs. 44.4%) in these patients. The age-related risk of complications and mortality associated with surgery have been addressed in several studies. In agreement with our data, advanced age was also found to be an important predictor of adverse outcomes in other patient populations^24–30^. Despite extensive efforts, mortality has not changed significantly in recent decades. In addition to the increasing frailty of the elderly population, the increased risk of surgery in the emergency setting, longer LOS and prolonged ventilation may explain the disproportionately high morbidity and mortality in elderly patients. However, because morbidity and surgical mortality are multifactorial, chronologic age should not be used as the sole criterion for determining the appropriateness of surgical intervention.

The rate of cardiac comorbidities was lower in the elective group than in the emergency group, and none of the cardiac comorbidities were independent predictors of in-hospital mortality. However, even after adjustment for other risk factors, PAD was an independent predictor of in-hospital mortality, implying that it has prognostic value not seen for other risk factors. This is consistent with data in the literature that show an association of PAD with an increased risk of mortality^31^.

As expected, the LOS was significantly longer in the elective group patients than in emergency group patients. This is not surprising because (1) all elective group patients underwent complex elective procedures^32^ with an average operation duration of 300 min. These patients would have been likely to have longer than average LOSs. This means that the occurrence of postoperative acute surgical complications requiring abdominal exploration inevitably extends the LOS beyond the expected discharge time. This is consistent with data reported in the literature that indicate a strong association between postoperative complications and adverse outcomes, including prolonged LOS^33,34^. Thus, the increase in LOS for patients with complications of elective surgery is in part attributable to the complexity of the initial elective surgical procedure and does not represent poor surgical standards. (2) Conversely, the outcome after EAE for primary high-risk emergencies is poor, and with a median of only 4 days between the index procedure and death, the survival time is short. Therefore, although the patients with complications of elective surgery stayed for a significantly longer time in the hospital than did those with primary emergencies, the difference was anticipated and attributable to the increased case-fatality rate within the first few days after surgery in the primary emergency group. Thus, while this difference between the two groups was statistically significant, its significance in real-world clinical practice is artificial.

The presented study has limitations that must be considered.

First, we relied on a retrospective data. As with any study using such data, there is a risk of miscoding and/or underreporting of clinical events such as complications. Second, the monocentric nature of our study limits the generalizability of the presented results. Third, the number of patients and the distribution of sex between groups were not well balanced. A more balanced patient cohort may have resulted in different attributable outcome profiles. Fourth, there were certain differences regarding comorbidities between groups. For example, regarding cardiac comorbidities, the emergency group was sicker than the elective group, but once a multivariate logistic regression analysis was completed, it would have adjusted for the difference. Finally, we controlled for available clinical confounders identified in our institutional database; however, there are certainly other confounders that we were unable to assess, which may have influenced the results.

Despite these limitations and given the clinical significance of the topic, our experience provides valuable information to clinicians and encourages future studies to expand on and further substantiate the findings.

## Conclusions

Patients undergoing EAE due to acute complications of major elective surgery tolerate the procedure relatively well compared with patients undergoing EAE due to primary major abdominal emergencies. The present study suggests that patient factors and excess medical complications contributed to the higher rate of mortality among high-risk abdominal emergency patients. The standard preoperative care provided by a multidisciplinary team in preparation for the elective surgical procedure may have contributed to the relatively favorable outcome in patients undergoing EAE due to complications of elective surgery. This suggests that preoperative optimization of patient factors, including appropriate cardiac and pulmonary evaluation and optimization (at least to the extent possible), is crucial for reducing morbidity and in-hospital mortality. The independent predictors of mortality obtained from the results of the present study have a discriminative ability to estimate the operative risk in high-risk surgical emergencies and may assist surgeons, patients and their families in decision-making.

## Supplementary Information

Supplementary Tables.

## Data Availability

The datasets generated and/or analyzed during the current study are not publicly available due to internal institutional restrictions but are available from the corresponding author on reasonable request and with the permission of the institution where the data were generated.
