# Supplementary material for: Assessing differences in surgical outcomes following emergency abdominal exploration for complications of elective surgery and high-risk primary emergencies
Source: Sci Rep. 2022 Jan 25;12:1349. doi: 10.1038/s41598-022-05326-4 (PMC8789789; doi:10.1038/s41598-022-05326-4)
Supplement: Supplementary file 1 — Supplementary Tables. [file 41598_2022_5326_MOESM1_ESM.docx]

Table S1 (Supplemental): Indications for Index emergency procedures

Indication Elective group Emergency group

N = 293 N = 776

Viscus organ perforation 40 (13.7) 294 (37.9)

Mesenteric ischemia 20 (6.8) 165 (21.3)

Hemorrhage 45 (15.4) 58 (7.5)

Bowel obstruction 16 (5.5) 208 (26.8)

Anastomotic leaks 81 (27.6) -

Bile leaks 25 (8.5) -

Pancreatitis 5 (1.7) 14 (1.8)

Burst abdomen 43 (14.7) -

Sepsis related to multiple abscesses 7 (2.4) 3 (0.4)

Abdominal compartment 5 (1.7) -

Laparotomy due to vascular events 2 (0.7) 21 (2.7)

Laparotomy related to traumatic events - 9 (1.2)

Complicated cholecystitis - 4 (0.5)

Numbers in bracket show values presented in n (%) unless noted otherwise.

Table S2 (Supplemental): procedure specific mortality rate (entire cohort; N = 1069)

Procedure Mortality

n (%)

Closure of viscus organ 177 (16.6) 54 (30.5)

Right colectomy including subtotal resection 122 (11.4) 53 (43.4)

Multivisceral procedures 144 (13.5) 60 (41.7)

Small bowel resection 127 (11.9) 45 (35.4)

Laparotomy with extensive adhesiolysis 197 (18.4) 42 (21.3)

Hartmann’s procedure 100 (9.4) 39 (39.0)

Hemostasis 82 (7.7) 34 (41.5)

Laparotomy only 29 (2.7) 26 (89.7)

Repair of intestinal perforation with formation of stoma 37 (3.5) 9 (24.3)

Vascular repair via laparotomy and bowel resection 16 (1.5) 9 (56.3)

Oesophagogastric resection of any type 14 (1.3) 9 (64.3)

Colectomy unspecified 18 (1.7) 5 (27.8)

Surgery for complicated cholecystitis 4 (0.4) 1 (25.0)

Splenectomy 2 (0.2) 0 (0.0)

Numbers in bracket show values presented in n (%) unless noted otherwise
